# Breed, sex and anatomical location-specific gene expression profiling of the porcine skeletal muscles

**DOI:** 10.1186/1471-2156-14-53

**Published:** 2013-06-15

**Authors:** Jie Zhang, Chaowei Zhou, Jideng Ma, Lei Chen, Anan Jiang, Li Zhu, Surong Shuai, Jinyong Wang, Mingzhou Li, Xuewei Li

**Affiliations:** 1 Institute of Animal Genetics & Breeding, College of Animal Science & Technology, Sichuan Agricultural University, Ya’an, Sichuan 625000, China; 2 Chongqing Academy of Animal Science, Chongqing 402460, China

**Keywords:** Gene expression, Microarray, Muscle, Pig

## Abstract

**Background:**

Skeletal muscle is one of the most important economic traits in agricultural animals, especially in pigs. In the modern pig industry, lean type pigs have undergone strong artificial selection for muscle growth, which has led to remarkable phenotypic variations compared with fatty type pigs, making these different breeds an ideal model for comparative studies.

**Results:**

Here, we present comprehensive gene expression profiling for the white (*longissimus dorsi* muscle) and the red (*psoas* major muscle) skeletal muscles among male and female fatty Rongchang, feral Tibetan and lean Landrace pigs, using a microarray approach. We identified differentially expressed genes that may be associated the phenotypic differences of porcine muscles among the breeds, between the sexes and the anatomical locations. We also used a clustering method to identify sets of functionally coexpressed genes that are linked to different muscle phenotypes. We showed that, compared with the white muscles, which primarily modulate metabolic processes, the red muscles show a tendency to be a risk factor for inflammation and immune-related disorders.

**Conclusions:**

This analysis presents breed-, sex- and anatomical location-specific gene expression profiles and further identified genes that may be associated with the phenotypic differences in porcine muscles among breeds, between the sexes and the anatomical locations.

## Background

Skeletal muscle is the most abundant tissue, comprising approximately 50% of the total body mass in mammals [1]. It is not only a motor organ, but also part of the endocrine system, participating in the regulation of whole body metabolism [2]. Skeletal muscle, as a highly heterogeneous tissue, is composed of a variety of functionally diverse myofibre types [3]; mainly the red (type I and IIa) and the white (type IIb) fibers. Red skeletal muscles, such as the *psoas* major muscles (PMM), have a higher percentage of capillaries, myoglobin, lipids and mitochondria [4], making them a better aerobic machine than the paler-appearing white muscle [5]. White skeletal muscles, such as the *longissimus doris* muscles (LDM) [4], are required for anaerobic glycolytic metabolism to support the high transient energy demand [6].

Deciphering the different gene expression patterns between the different tissues would aid in our understanding of their distinct metabolic features. Mo *et al.* identified various candidate genes involved in cell adhesion, energy balance, muscle atrophy and myogenesis by comparing patterns of gene expression in three independent mouse models of Kennedy disease/spinal bulbar muscular atrophy [7]. Wolfs *et al.* reported that coexpressed immune and metabolic genes are associated with plasma high density lipoprotein and glucose levels by comparing genome-wide transcription profiling of subcutaneous and visceral adipose tissues obtained from obese patients [8]. Previous reports also suggested that ethnic group and sex are also the important factors that affect physiological and biochemical features of skeletal muscles in mammals [9-12].

Pigs are important agricultural animals and ideal biomedical models [13]. In the modern pig industry, pigs have undergone strong artificial selection for lean meat or adipose production, which has led to remarkable phenotypic variations, making these different breeds a perfect model for comparative studies [14,15]. Using a microarray approach, Bai *et al.* noted that most differentially expressed genes between porcine PMM and LDM were of mitochondrial origin [16]. Li *et al.* (2010) reported that the differentially expressed genes between the LDM and *soleus* muscle of Chinese Meishan pigs were mainly over-represented in various signaling pathways (particularly TGF-β, MAPK, Wnt, mTOR and insulin pathways) [17]. Nonetheless, the different gene expression profiles associated with breed and sex in skeletal muscle tissues has been long overdue, and elucidation of this information will benefit the development of strategies for skeletal muscle manipulation.

Here, using a microarray technology, we present a comprehensive survey of gene expression profiles between two phenotypically distinct skeletal muscles and sexes of three well-defined pig breeds displaying distinct muscle phenotypes. This study will contribute to our understanding of the molecular process of muscle fiber type formulation and provide a theoretical basis for breed and meat quality improvement in pigs.

## Results and discussion

### Phenotypic measurements

Our previous report, based on the same individuals, demonstrated that the myofibre cross-sectional area (CSA) and myofibre ratio were significant different between the two skeletal tissues, between the male and female and among the three breeds [18] (Additional file 1: Figure S1). In addition, 24 representative metabolism indicators in serum also revealed the same ranking from the leaner Landrace, the wild Tibetan and the fatty Rongchang pigs [18] (Additional file 2: Table S1).

### Functional enrichment analysis of differentially expressed genes

**Figure 1:**
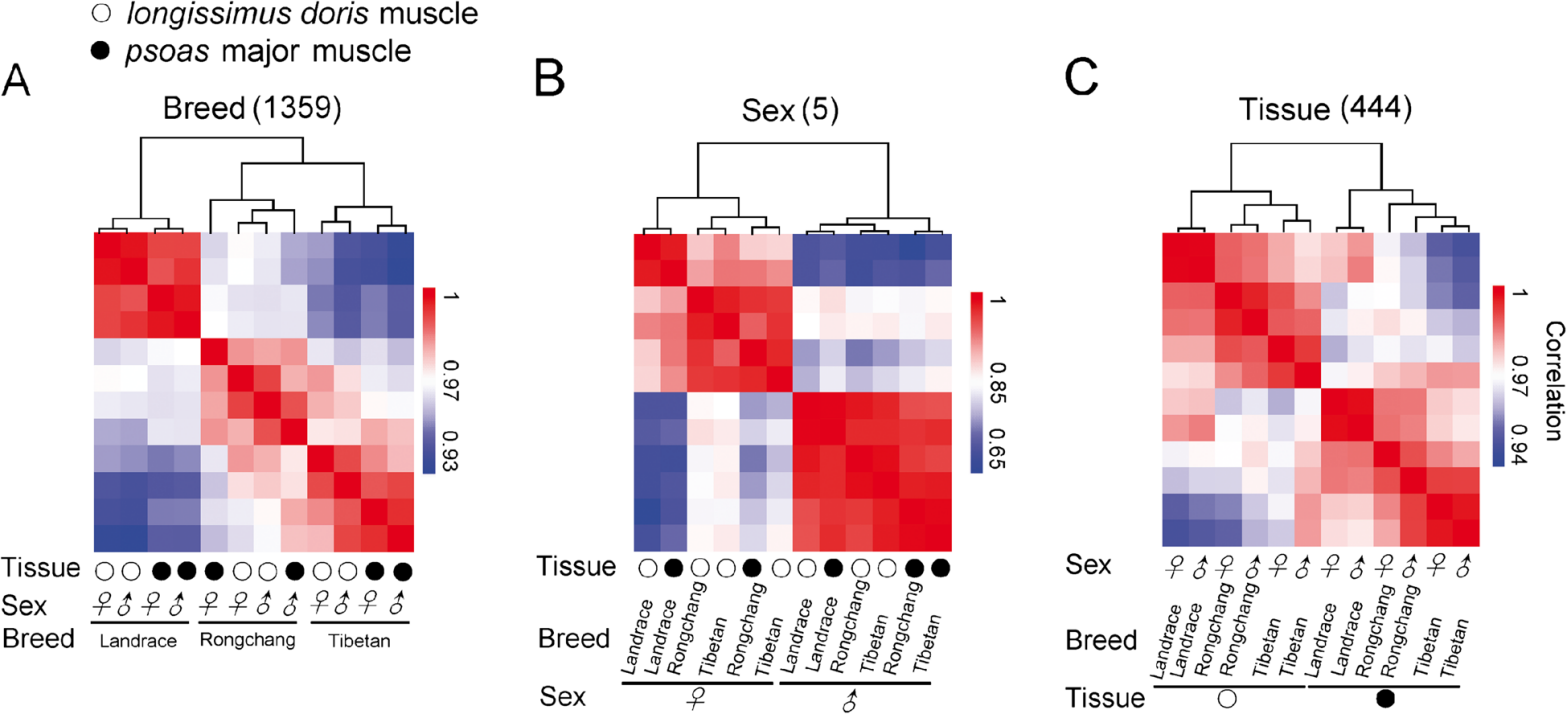
**Heat map matrix of Spearman correlations among samples. (A)** The counts of 1,359 DEGs among the three breeds. **(B)** The counts of five DEGs between the sexes. **(C)** The counts of 444 DEGs between the two tissues.

Out of 4,309 high-confidence and well annotated probe-targeted genes (Additional file 3: Tables S2), we identified five (0.12%), 444 (10.3%) and 1,359 (31.54%) differentially expressed genes (DEGs) between the sexes and the two tissues, and among the three breeds (*P* < 0.05, three-way ANOVA, *n* = 3 per breed per sex per tissue) (Additional file 4: Tables S3), respectively. These DEGs could discriminate the different breeds, sexes and tissues (Figure 1). The high number of DEGs among three pig breeds implies distinct muscle features among different pig breeds. In addition, the biological replicates correlated with each other (average Spearman’s *r* = 0.99, Figure 1), which suggested experimental reliability and further highlighted the low variation in gene expression profiles across different individuals.

**Figure 2:**
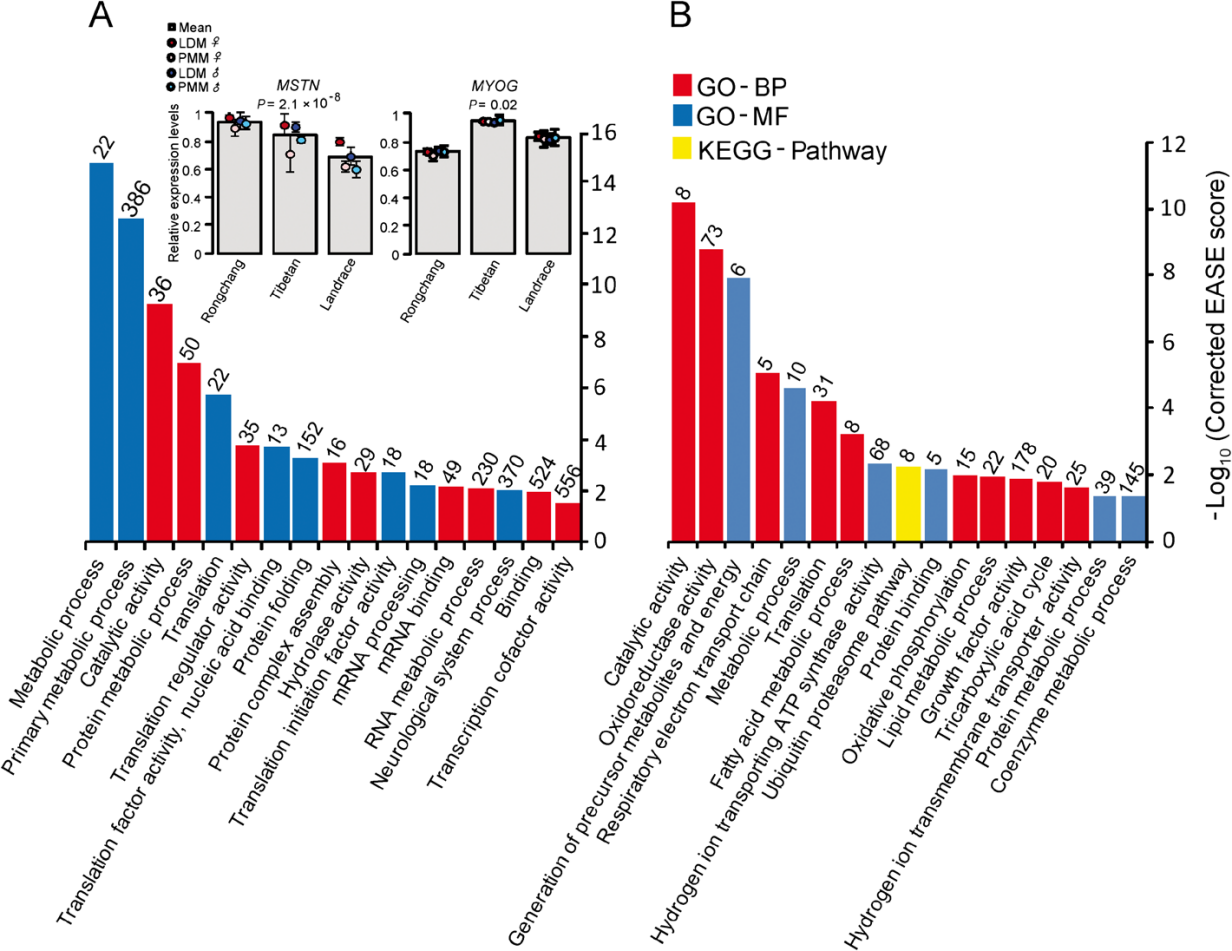
**Analysis of DEGs. (A)** Gene Ontology (GO) categories enriched for breed-specific DEGs and relative expression level of *MSTN* and *MYOG* genes involved in breed-specific DEGs. LDM and PMM mean *longissimus dorsi* muscle and *psoas* major muscle, respectively. Datas are means ± SD. The significance of differences among samples was determined by One-way ANOVA. **(B)** Gene Ontology (GO) categories enriched for tissue-specific DEGs. The EASE score, which indicated the significance of the comparison, was calculated by Benjamini-corrected modified Fisher’s exact test. BP, biological process; MF, molecular function.

We found that the breed-specific DEGs were significantly enriched in the Gene Ontology (GO) categories of protein metabolism (i.e. protein metabolic process, translation, protein folding and protein complex assembly) and RNA metabolism (i.e. mRNA processing and RNA metabolic process) (Figure 2A). Various well-known genes involved in growth and development of skeletal muscles were identified. For example, myostatin (*MSTN*), a secreted transforming growth factor (TGF) beta protein family member, inhibits the differentiation and growth of muscle and Akt-induced protein synthesis [19]. The expression level of *MSTN* was highest in Rongchang pigs and lowest in Landrace pigs, which is consistent with the breeds’ characteristics (Figure 2A). Myogenin (*MYOG*) transforms potential mesoderm cells to sarcoblasts, and has a critical role in the terminal differentiation of the specified muscle cells [20,21]. Among the three breeds, the expression levels of *MYOG* were highest in Tibetan pigs and lowest in Rongchang pigs (Figure 2A). This result suggests that the breed-specific differences in muscle were mainly related to the protein translation process, which is consistent with previous studies [22-24]. Additionally, we found breed-specific DEGs that were over-represented in the neurological system process (370 DEGs, *P* = 0.01), which highlights the important roles of myoblast lineage and innervations in the diversification of skeletal muscle fiber types.

Tissue-specific DEGs were significantly enriched in energy metabolism related processes (i.e. generation of precursor metabolites and energy, respiratory electron transport chain, fatty acid metabolic process, oxidative phosphorylation, lipid metabolic process, tricarboxylic acid cycle and coenzyme metabolic process) (Figure 2B), which is consistent with the distinct features of energy expenditure regulation between the LDM and PMM [25]. Energy availability is important in the formation of mature muscle fibers and is essential for muscle proliferation and differentiation. Louis *et al.* reported that the energy content of cultured satellite cells is related to the hypertrophy of myofibres *in vitro,* which indicated a direct connection between energy metabolism and myogenesis [26]. Cagnazzo *et al.* also demonstrated that myogenic differentiation and energy metabolism were directly connected processes [27]. Genes involved in energy metabolism were identified. For example, *MDH1*, *PDK3* and *GOT1* play important roles in sympathetic-induced metabolism, which is involved in modulating the activity of glyceroneogenesis [28]. *MDH1*, *PDK3* and *GOT1* showed lower gene expression levels in the LDM than in PMM (Additional file 5: Figure S2), which agreed with previous reports [29-32]. We also found that tissue-specific DEGs were over-represented in the ubiquitin-proteasome pathway (Figure 2B), which plays a critical role in the adaptation of skeletal muscle to persistent decreases or increases in muscle activity. The ubiquitin-proteasome pathway is constitutively active in muscle and continually regulates protein turnover [33].

**Figure 3:**
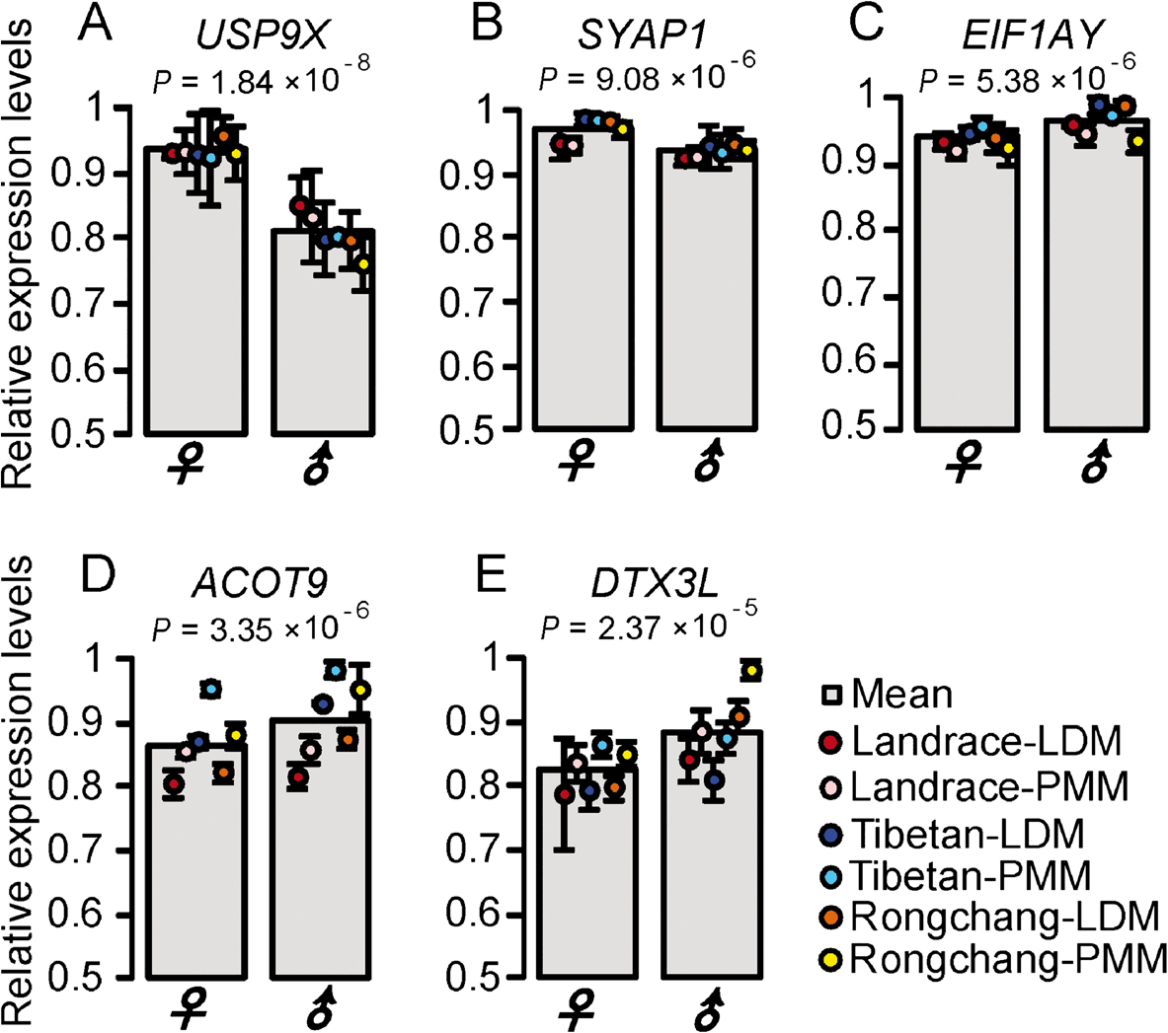
**Relative expression levels of sex-specific DEGs. (A)***USP9X* gene; **(B)***SYAP1* gene; **(C)***EIF1AY* gene; **(D)***ACOT9* gene; **(E)***DTX3L* gene. LDM and PMM mean *longissimus dorsi* muscle and *psoas* major muscle, respectively. Data are means ± SD. The significance of differences among samples was determined by Student’s *t*-test.

We only identified five DEGs between the sexes, of which two are X-linked genes (ubiquitin specific peptidase 9 (*USP9X)* and synapse associated protein 1 *(SYAP1*)) that exhibited higher expression levels in females than in males (*P* < 10^-5^, Student’s *t*-test; Figure 3A and Figure 3B). *USP9X*, as a novel mTORC1 and −2 binding partner, negatively regulates mTOR activity and further affects the differentiation of skeletal muscle [34]. *SYAP1* plays an important role in cancer formation [35]. By contrast, a Y-linked gene, eukaryotic translation initiation factor 1A (*EIF1AY*) exhibited significantly higher expression in males than in females (*P* = 5.38 × 10^-6^, Student’s *t*-test; Figure 3C), which could affect the maximal rate of protein biosynthesis [36]. Additionally, two DEGs are located in the autosome: acyl-CoA thioesterase 9 (*ACOT9*) and the deltex 3-like (*DTX3L*), which exhibited higher mRNA expression levels in males than in females (*P* < 10^-4^, Student’s *t*-test; Figure 3D and Figure 3E). *ACOT9*, as an important enzyme involved in fatty acid metabolism, is located in the mitochondrion and provides energy through the citric acid cycle [37]. The higher mRNA expression level of *ACOT9* in males reflects the fact that male muscles have a higher capacity for anaerobic metabolism and generate a higher maximum power output than female muscles [11]. *DTX3L* plays an important role in the Notch signaling pathway and controls myogenesis; its higher expression in male muscles is consistent with male pigs having more and larger muscles than the females [38].

### Validation of gene expression changes by Quantitative PCR (Q-PCR)

Six genes (*ADIPOR1*, *ADIPOR2*, *CAV1*, *CAV2*, *INSIG1*, and *MDH1*) were selected to confirm their expression patterns using Q-PCR. The results indicated that the expression patterns of these genes were consistent with the microarray (average Pearson’s *r* = 0.86; Additional file 6: Figure S3).

### Analysis of coexpressed gene modules

To extract more biological information within the genome-wide expression data set that could not be provided by individual, we constructed coexpressed gene modules and performed association analysis with the phenotypic traits, as did previous reports [8].

**Figure 4:**
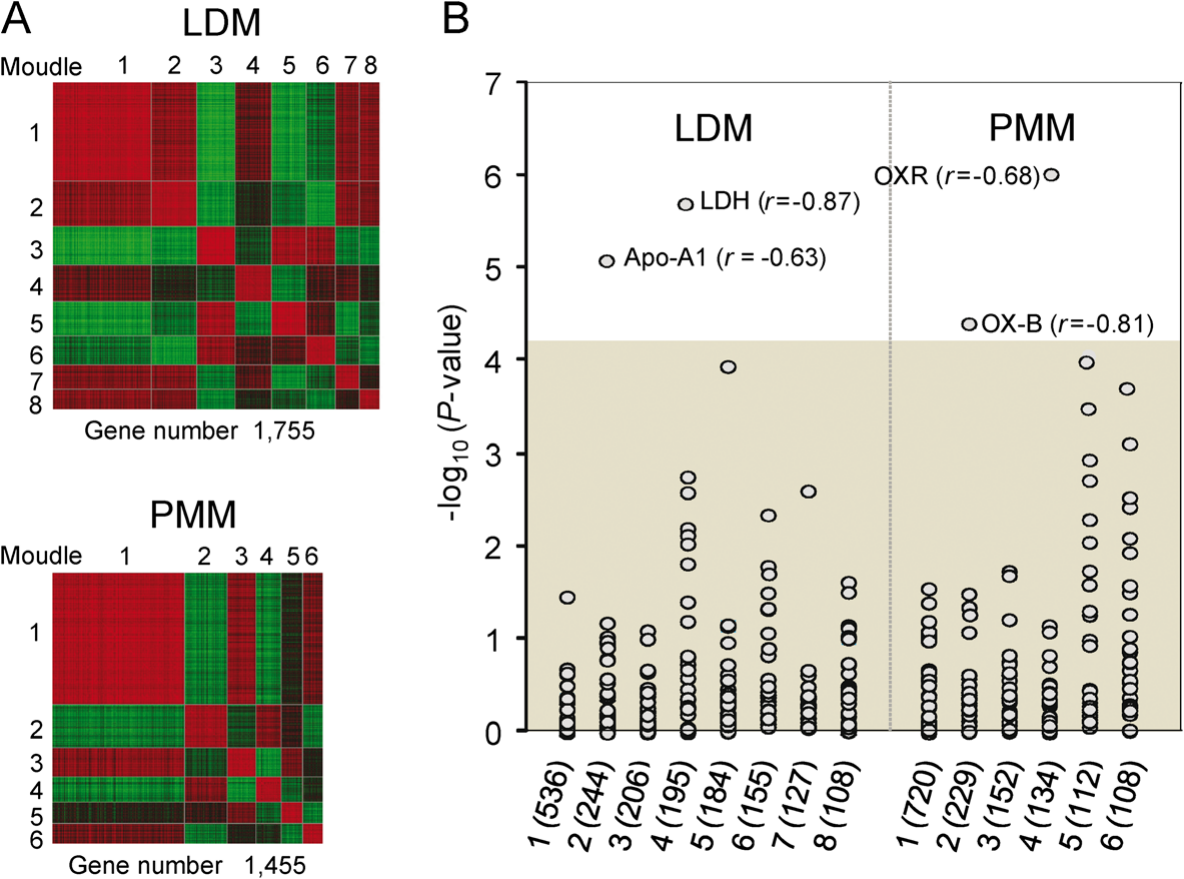
**Analysis of coexpressed gene modules in LDM and PMM. (A)** Heat map of coexpressed gene modules in *longissimus dorsi* muscle (LDM) and *psoas* major muscle (PMM). Gene pairs strongly positively or negatively correlated are shown in red or green, respectively. **(B)** Correlations between coexpressed gene modules in two muscle tissues and phenotypic traits. -Log *P*-values for Spearman correlation coefficients between the values of the modules and the different phenotypic traits are shown. The gray shadow represents a highly stringent Bonferroni corrected *P*-value of 0.05.

We identified eight and six gene modules for LDM and PMM (more than 100 genes per module), representing 1,755 and 1,455 genes, respectively (Additional file 7: Table S4A and 4B). Expressions of genes within a single gene module are strongly correlated, whereas genes that belong to different modules generally show no significant coexpression (Figure 4A). As shown in Additional file 8: Table S5, eight gene modules of LDM and PMM significantly overlapped with each other (*P* < 0.01, Fisher’s exact tests), which implies that similar gene expression patterns are involved in basic physiological and biochemical processes of skeletal muscle.

We identified two coexpressed gene modules in LDM that were significantly negatively correlated with the amount of apolipoprotein A1 (Apo-A1) (Spearman’s *r* = −0.63, *P* =8.71 × 10^-6^) and lactate dehydrogenase (LDH) (Spearman’s *r* = −0.87, *P* =2.05 × 10^-6^, Figure 4B) in serum, which are primarily involved in metabolic processes. Apo-A1 is a major protein component of high density lipoprotein in serum and has been suggested to be tightly linked to muscle differentiation [39]. LDH is a marker of the oxidative and glycolytic capacities of the muscle, and converts pyruvate to lactate when oxygen is absent or in short supply [40]. The genes within these two gene modules were mainly enriched in the categories of protein metabolic process (94 genes, *P* = 5.03 × 10^-4^), cellular metabolic process (100 genes, *P* = 5.22 × 10^-6^), cellular nitrogen compound metabolic process (60 genes, *P* = 0.048) and primary metabolic process (127 genes, *P* = 4.55 × 10^-8^) (Table 1). These findings confirmed the report that the LDM is mainly associated with metabolic rate [41].

**Table 1:** Gene Ontology (GO) categories enriched for coexpressed gene modules that correlated with phenotypic traits

**Tissues (gene module no.)**	**Correlated trait**	**Functional category**	**Term description**	***P *value**	**Involved gene no.**
LDM (2)	Apo-A1	GO-BP	Metabolic process	1.06 × 10^-7^	139
		GO-BP	Primary metabolic process	4.55 × 10^-8^	127
		GO-BP	Protein metabolic process	5.03 × 10^-4^	94
		GO-BP	Carbohydrate metabolic process	0.028	82
		GO-BP	Protein modification process	0.016	73
		GO-BP	Protein amino acid phosphorylation	0.016	65
LDM (4)	LDH	GO-BP	Cellular metabolic process	5.22 × 10^-6^	100
		GO-BP	Metabolic process	5.58 × 10^-5^	105
		GO-BP	Cellular process	2.47 × 10^-4^	128
		GO-BP	Cellular nitrogen compound metabolic process	0.048	60
		GO-BP	Primary metabolic process	0.016	93
PMM (2)	OX-B	GO-BP	Immune system process	2.12 × 10^-5^	29
		GO-BP	Inflammatory response	0.001	16
		GO-BP	Response to external stimulus	0.001	26
		GO-BP	Response to wounding	0.001	20
		GO-BP	Immune response	0.001	22
		GO-BP	Regulation of immune system process	0.002	16
		GO-BP	Regulation of response to stimulus	0.004	17
		GO-BP	Positive regulation of immune system process	0.006	12
		GO-BP	Regulation of immune response	0.012	12
		GO-BP	Lymphocyte activation	0.021	11
		GO-BP	Cell activation	0.031	12
		GO-BP	Leukocyte activation	0.033	11
		GO-BP	Positive regulation of lymphocyte activation	0.049	8
		GO-MF	Receptor binding	2.19 × 10^-5^	30
PMM (4)	OXR	GO-BP	Immune response	0.019	9
		GO-BP	Immune system process	1.79 × 10^-6^	16
		GO-BP	Cellular defense response	0.027	6
		GO-BP	Response to stimulus	4.87 × 10^-4^	12
		GO-BP	Cell-matrix adhesion	2.24 × 10^-3^	10

We also found that two coexpressed gene modules in PMM were significantly negatively correlated with amount of orexin-B (OX-B) (Spearman’s *r* = −0.81, *P* =5.75 × 10^-5^) and the orexin receptor (OXR) (Spearman’s *r* = −0.68, *P* = 1.04 × 10^-6^, Figure 4B) in serum, which are representative indicators for the inflammatory process and the immune system in serum. The genes within these two gene modules were mainly enriched in the categories of the immune system process (29 genes, *P* = 2.12 × 10^-5^), inflammatory response (16 genes, *P* = 0.001), immune response (22 genes, *P* = 0.001), lymphocyte activation (11 genes, *P* = 0.02), leukocyte activation (11 genes, *P* = 0.03), and cellular defense response (6 genes, *P* = 0.02) (Table 1), which suggests that the PMM is a metabolic risk factor. This finding is consistent with evidence that shows that the PMM is supplied by venous blood from the lumbar spine and has lymphatics overlying the muscle from nearby intra-abdominal organs, making it highly susceptible to contiguous infection and inflammation from organs such as the colon, appendix, terminal ileum and several intra-abdominal structures [42-44].

## Conclusions

The analysis presented the gene expression profiles and identified DEGs that may be related to the phenotypic differences in porcine muscles among breeds, between the sexes and the anatomical locations. The results provide a basis for further exploration of the molecular process of muscle fiber type formulation, and may also help the further development of biomarkers for important economic traits (i.e. pork quality and yield) in pigs.

## Methods

### Sample preparation

Three females and three males at 210-days-old for each of the leaner Landrace pigs, the wild Tibetan pigs and the fatty Rongchang pigs were used in this study as previously described [18]. Animals were humanely sacrificed, according to the Regulations for the Administration of Affairs Concerning Experimental Animals (Ministry of Science and Technology, China, revised in June 2004) and approved by the Institutional Animal Care and Use Committee in the College of Animal Science and Technology, Sichuan Agricultural University, Sichuan, China. The *longissimus dorsi* muscle (LDM, typical white muscle) near the last 3^rd^ or 4^th^ rib and the intermediate section of *psoas* major muscle (PMM, typical red muscle) were rapidly separated from each carcass. Samples were frozen in liquid nitrogen, and stored at −80°C until RNA extraction. For more information, please refer to Li *et al*. [18].

### Measurements of skeletal muscle-related phenotype

Measurements of concentrations of 24 serum-circulating indicators of metabolism, myofibre cross-sectional area and myofibre ratio (type I vs. II) are from our previous report based on same individuals. For more information, please refer to Li *et al*. [18].

### Extraction of RNA

Total RNA was extracted from 36 samples using TRIzol (Invitrogen). RNA was purified and DNase treated using an RNeasy column (Qiagen) according to the manufacturer’s instructions. The quantity of each RNA sample was examined by the NanoDrop ND-1000 spectrophotometer (Nano Drop) at 260/280 nm (ratio > 2.0). The integrity of total RNA also passed analysis with the Bioanalyzer 2100 and RNA 6000 Nano LabChip Kit (Agilent Technologies) with RIN number > 6 (7.6 ± 0.3, n = 36).

### Microarray analyses

Agilent Oligo microarrays were used to determine global gene expression of 36 samples. Individual microarrays were performed for each sample. Hybridization, washing, and scanning were done according to standard Agilent protocols. Generated array images were loaded into Feature Extraction Software (Agilent Technologies) for feature data extraction, and data analysis was performed with MultiExperiment Viewer (MeV) [45]. Array data have been uploaded to NCBI’s Gene Expression Omnibus (GEO) [accession number GSE30343]. For more information, please refer to Li *et al.*[18].

To obtain high-confidence gene expression data, we mapped 43,603 probes (60 mer in length) to the pig reference genome allowing up to one mismatch, and further filtered unannotated pig target sequences which resulting 4,309 genes were used in subsequent analysis. (Tables S2). To identify differentially expressed mRNAs (*P* < 0.05) for the clustering analysis, we used three-way ANOVA for comparisons. Resulting *P-*values of above tests were corrected with adjusted Bonferroni method (FDR < 0.01, 1,000 permutations).

### Construct modules of coexpressed genes

For LDM and PMM separately, modules of highly coexpressed genes were constructed using pair wise average-linkage cluster analysis as previously described [8,46]. We kept repeating this as an iterative process until the most significantly correlated pair was *r* < 0.8. To visualize the correlations between probes within the modules, we constructed colored heatmaps by plotting pair-wise correlation values of expression of all the probes within the modules. To calculate significance of overlap in gene content between modules and between different datasets, we performed Fisher’s exact tests.

### Function enrichment analysis of genes

To elucidate the biological mechanisms associated with the genes that are correlated to the phenotypic traits, we performed functional enrichment analysis of Gene Ontology (GO) for genes using DAVID software [47].

### Quantitative PCR (Q-PCR)

We selected six genes randomly to validation experiment using Q-PCR. Primer sequences used for the Q-PCR are shown in Additional file 9: Table S6. Porcine *ACTB*, *TBP* and *TOP2B* were simultaneously used as endogenous control genes [48]. Relative expression levels of objective mRNAs were calculated using the ΔΔCt method.

## Competing interests

The authors have declared that no competing interests exist.

## Authors’ contributions

MZL and XWL conceived and designed the experiment. JZ and MZL performed the data analysis and drafted the manuscript. AAJ, LZ, SRS and JYW collected the samples, statistical analysis and prepared nucleic acids. CWZ, JDM and LC performed gene expression microarray. All authors read and approved the final manuscript.

## Supplementary Material

Additional file 1: Figure S1 The differences of the (A) myofibre CSA and (B) myofibre ratio among samples. Data are means ± SD. The significance of differences among samples was determined by the three-way ANOVA; B, S and T refer to the breed, sex and tissue, respectively. Click here for file

Additional file 2: Table S1 Serum parameters of the study population. Datas are means ± SD. Total cholesterol (TC), triglycerides (TG), high density lipoprotein (HDL), low density lipoprotein (LDL), very-low density lipoprotein (VLDL), lactate dehydrogenase (LDH), apolipoprotein A-1 (Apo-A1), apolipoprotein B (Apo-B), adiponectin (Adipo), adiponectin receptor (AdipoR), C-peptide, cholecystokinin (CCK), gastrin receptor (GsaR), growth hormone (GH), highly sensitive C-reactive protein (hs-CRP), insulin, interleukin - 6 (IL-6), leptin (Lep), leptin receptor (LepR), orexin-B (OX-B), orexin receptor (OXR), plasminogen activator inhibitor-1 (PAI-1), tumor necrosis factor-α (TNF-α) and somatostatin (SS). Click here for file

Additional file 3: Table S2 Complete gene list used analysis (annotated genes only). Click here for file

Additional file 4: Table S3 Differential expressed genes among the (A) breeds, between the (B) sexes and the (C) anatomical locations. Click here for file

Additional file 5: Figure S2 Genes involved in tissue-specific DEGs. Datas are means ± SD, Student’s *t*-test; LDM and PMM refer to the *longissimus doris* muscle and *psoas* major muscle, respectively. Click here for file

Additional file 6: Figure S3 Validation of gene expression by Q-PCR. The data presented in Y-axis indicated the relative mRNA expression of both microarray and Q-PCR. Datas are means ± SD. The Pearson correlation coefficient (*r*) and the corresponding significance value (*P*) were shown above the columns. Click here for file

Additional file 7: Table S4 Contents of genes in module generated in (A) *longissimus doris* muscle (LDM) and (B) *psoas* major muscle (PMM). For each module, the number genes and the names of all those genes are listed. Click here for file

Additional file 8: Table S5 Overlap between genes in the modules identified in *longissimus doris* muscle (LDM) and *psoas* major muscle (PMM). *P*-values to determine the significance of the overlap between the modules were performed using a Fisher’s exact test: *1: *P* = 1.45 × 10^-10^; *2: *P* = 3.39 × 10^-12^; *3: *P* = 3.29 × 10^-18^; *4: *P* = 3.38 × 10^-18^; *5: *P* =1.09 × 10^-2^; *6: *P* = 7.24 × 10^-15^; *7: *P* = 9.52 × 10^-5^; *8: *P* = 1.62 × 10^-4^. Click here for file

Additional file 9: Table S6 Primer sequences used for Q-PCR. *: *ACTB* (β actin), *TBP* (TATA box binding protein) and *TOP2B* (topoisomerase II β) are the endogenous control genes. Click here for file
