# Supplementary figures and images for: Breed, sex and anatomical location-specific gene expression profiling of the porcine skeletal muscles
Source: BMC Genet. 2013 Jun 15;14:53. doi: 10.1186/1471-2156-14-53 (PMC3703266; doi:10.1186/1471-2156-14-53)

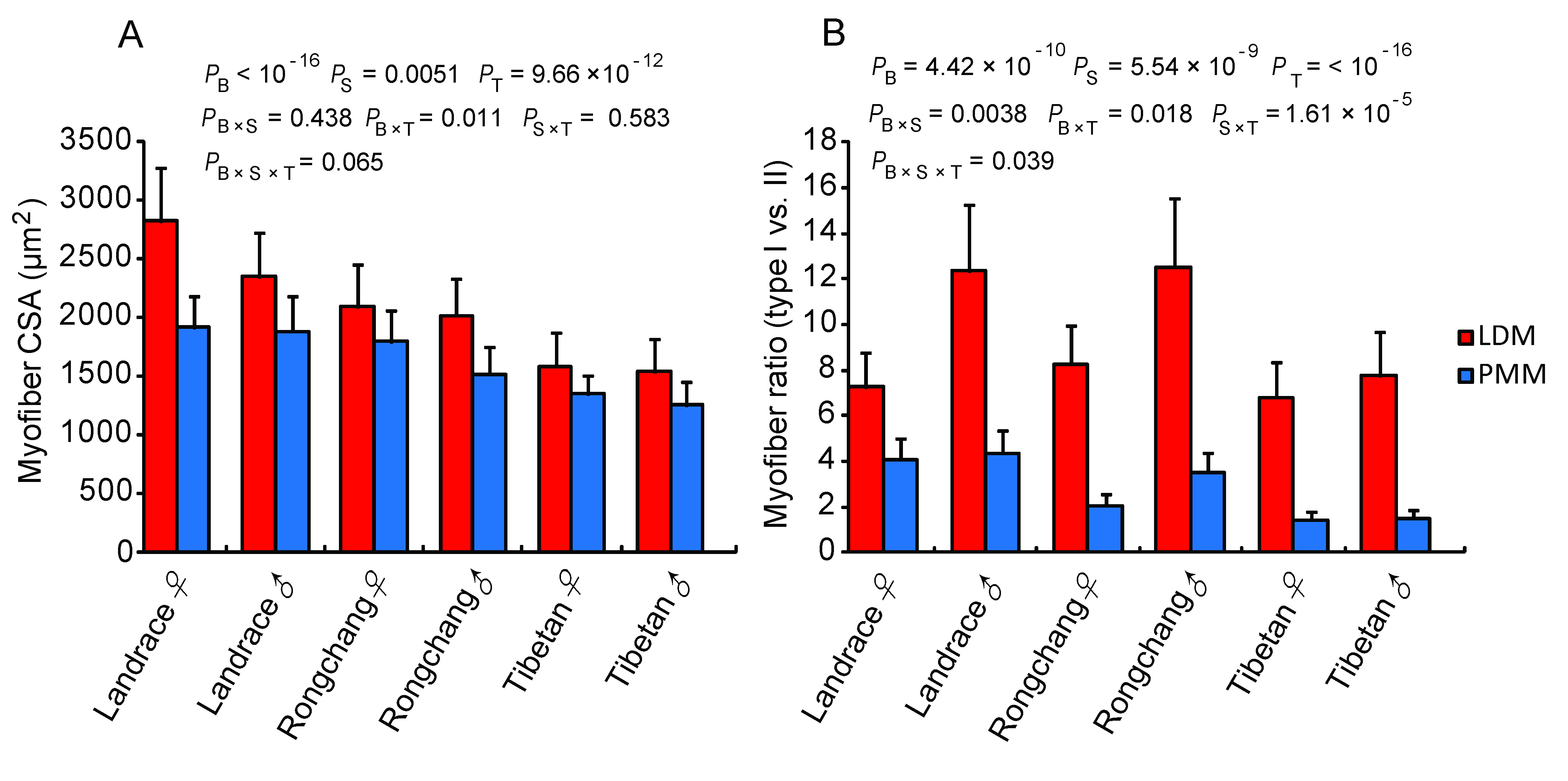

Supplement: Additional file 1: Figure S1 — The differences of the (A) myofibre CSA and (B) myofibre ratio among samples. Data are means ± SD. The significance of differences among samples was determined by the three-way ANOVA; B, S and T refer to the breed, sex and tissue, respectively. [file 1471-2156-14-53-S1.tiff]

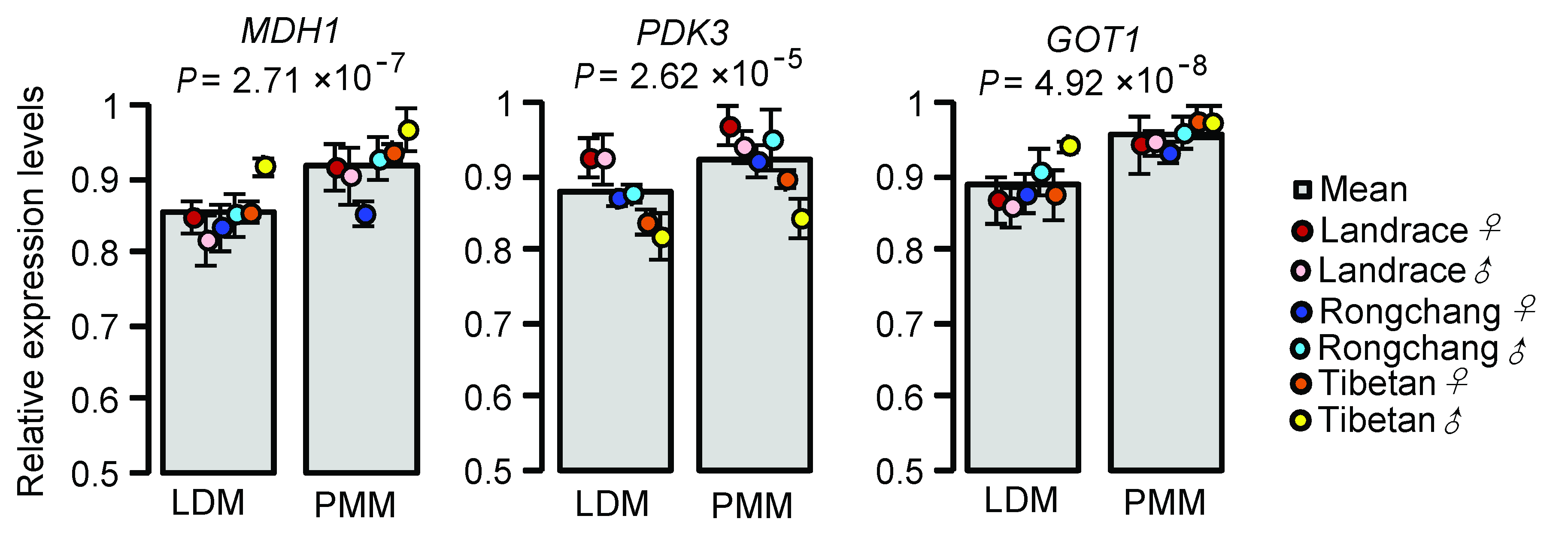

Supplement: Additional file 5: Figure S2 — Genes involved in tissue-specific DEGs. Datas are means ± SD, Student’s t-test; LDM and PMM refer to the longissimus doris muscle and psoas major muscle, respectively. [file 1471-2156-14-53-S5.tiff]

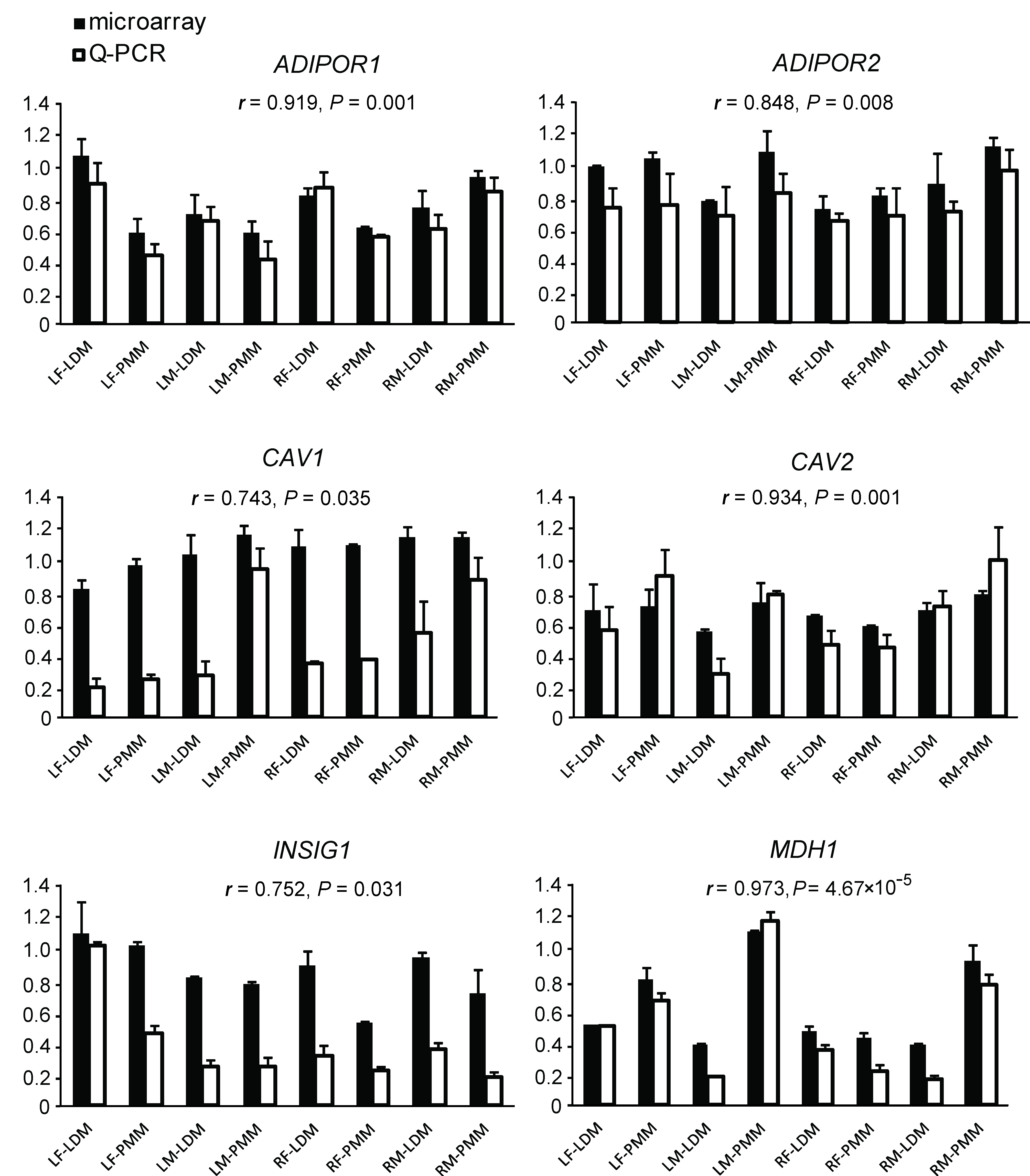

Supplement: Additional file 6: Figure S3 — Validation of gene expression by Q-PCR. The data presented in Y-axis indicated the relative mRNA expression of both microarray and Q-PCR. Datas are means ± SD. The Pearson correlation coefficient (r) and the corresponding significance value (P) were shown above the columns. [file 1471-2156-14-53-S6.tiff]
